# Recent advances and research progress regarding monoclonal antibodies for chronic graft-versus-host disease

**DOI:** 10.1016/j.heliyon.2024.e38460

**Published:** 2024-09-25

**Authors:** Shiqin Huang, Xianjing Cheng, Guancui Yang, Ruihao Huang, Yimei Feng, Lingyu Zeng, Tao Wu, Qingxiao Song, Xiaoqi Wang, Xi Zhang

**Affiliations:** a Medical Center of Hematology, Xinqiao Hospital of Army Medical University, Chongqing 400037 China; b Chongqing Key Laboratory of Hematology and Microenvironment, Chongqing 400037 China; c State Key Laboratory of Trauma and Chemical Poisoning, Army Medical University, Chongqing 400037, China; d Jinfeng Laboratory, Chongqing 400000, China; e Blood Diseases Institute, Xuzhou Medical University, Department of Hematology, The Affiliated Hospital of Xuzhou Medical University, Xuzhou 221002, Jiangsu, China; f Department of Hematology, Center of Hematologic Diseases of Chinese PLA, Lanzhou Military Command General Hospital, Lanzhou 730050, Gansu, China

**Keywords:** HSCT, Chronic GVHD, Monoclonal antibody

## Abstract

Chronic graft-versus-host disease (cGVHD) is one of the leading causes of mortality following allogeneic hematopoietic stem cell transplantation (HSCT), with only 50 % of patients responding to conventional corticosteroids with or without calcineurin inhibitors. Monoclonal antibodies (mAbs), such as CD20 mAbs, are the first drugs to demonstrate greater efficacy than corticosteroids in first-line treatment of cGVHD. This review provides a comprehensive overview of recent developments in the clinical utilization of mAbs to prevent and treat cGVHD. We also describe the application of drugs with target sites that are identical to or linked to those of cGVHD in autoimmune diseases as potential future treatments for cGVHD. In summary, we collected known evidence concerning the clinical research progress on the use of mAbs in the prevention and treatment of cGVHD and identified drugs with the same target sites in autoimmune diseases for potential future applications in cGVHD, with the goal of providing suggestions and inspiration for optimizing cGVHD treatment strategies and research directions.

## Highlights

• MAbs are highly homogeneous antibodies produced by B-cells that target only a specific epitope.
• MAbs can enhance the therapeutic potential of nonoverlapping pathways, providing therapeutic prospects for the immunocompromised, toxic and disabling effects of cGVHD.
• Monoclonal antibodies act locally while affecting the whole body, maintaining immune response, allowing the reduction or even cessation of immunosuppressive therapy, and improving or even reversing organ-specific fibrosis, thereby reducing the burden of symptoms and improving quality of life.

## 1 Introduction

Allogeneic hematopoietic stem cell transplantation (allo-HSCT) is the only potential cure for many high-risk hematological malignancies, and an increasing number of patients have benefited from this treatment and achieved a longer life expectancy [1,2]. However, chronic graft-versus-host disease (cGVHD) is one of the most common complications after HSCT. This condition occurs in 30 %–50 % of patients who undergo allo-HSCT and remains the leading cause of late-onset and nonrelapse mortality (NRM) [3,4]. Furthermore, severe cGVHD following allo-HSCT is debilitating and substantially impacts the quality of life (QoL) and functional status of survivors [5]. Systemic corticosteroids (CSs) are used as first-line treatments for moderate to severe cGVHD. Unfortunately, only approximately 50 % of patients respond to CSs treatment, and the response is usually partial and not persistent; more than half of patients require second-line therapy within 2 years [6]. Concerns have been raised regarding the irreversibility of damage to specific organs and the occurrence of exacerbations of cGVHD during steroid withdrawal [7]. In addition, long-term use of immunosuppressants, including steroids, is associated with substantial toxicity and an increased risk of infection [8]. Some newer therapeutic agents, such as the JAK1/2 inhibitors ruxolitinib and ibrutinib, show wide variations in efficacy in different organs and are associated with many side effects [9,10]. Together with the diverse clinical manifestations of cGVHD and recalcitrant fibrosis, these issues indicate the major difficulties in treatment of this condition. Therefore, innovative therapeutic strategies need to be developed.

As the understanding of the pathophysiology of GVHD continues to evolve, new therapeutic modalities are being translated to clinical practice to improve traditional immunosuppressive strategies, with therapeutic models focused more on inhibiting B-cell activation and priming in addition to T-cell targeting in cases of steroid-resistant cGVHD (SR-cGVHD) [11].

The development of monoclonal antibodies (mAbs) that target specific lymphocyte subsets without affecting other cells is a promising strategy for the clinical treatment of cGVHD. mAbs, which are highly homogeneous antibodies produced by the cloning of a single B-lymphocyte via genetic engineering techniques and directed only against a specific antigenic epitope, have been developed based on an understanding of the pathogenesis of a specific disease and the mode of action of specific proteins and molecules in the disease-causing process and belong to a class of targeted drugs [12,13].

The addition of rituximab to CSs as the first-line treatment for newly diagnosed cGVHD was associated with an 83 % objective response rate (ORR) at one year and was demonstrated to be safe and effective in a phase II prospective multicenter trial[14]. The combination of rituximab and corticosteroids as the first-line treatment for cGVHD is a new strategy that challenges previous standard first-line treatments.

**Fig. 1:**
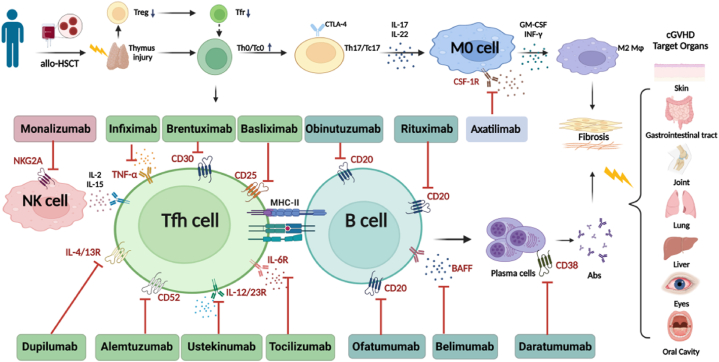
mAbs target in the treatment of cGVHD. cGVHD microscopically manifests early inflammation caused by tissue damage, thymus damage and dysregulation of T cells and B cells, and fibrosis caused by antibody deposition, which can also coexist with other stages.Macroscopically, it is manifested as damage to the main target organs of the skin, gastrointestinal tract, joint, lung, liver, eyes and oral cavity.Monoclonal antibody drugs targeting B cells, T cells and cytokines are precisely targeted to block the occurrence and development of cGVHD: anti-CD20 rituximab/ofatumumab/obinutuzumab, anti-CD38 daratumumab, anti-CD52 alemtuzumab, anti-CD30 brentuximab, anti-CD25 basliximab, anti-IL-6R tocilizumab, anti-TNF-α infiximab, anti-CSF-1R axatilimab, anti-IL12/23R ustekinumab, anti-NKG2A monalizumab, anti-BAFF belimumab and etc.

This review focuses on recent advances and research progress regarding mAbs for cGVHD, including preclinical studies and clinical studies for the prevention and treatment of this condition through various cellular targets, and we also identify drugs affecting same targets in preclinical studies of autoimmune diseases as potential future drugs for treating cGVHD. Table 1, Table 2 summarize recent advances in clinical studies of mAbs for the prevention and treatment of cGVHD. Table 3 outlines an overview of the drugs focused on different interventions at different clinical stages; the mechanisms of action of the drugs are succinctly delineated in Fig. 1.

**Table 1:** The applications of mAbs in prevention cGVHD.

Ref	Year	Study Type^a^	Conditioning^b^ N(%)	Donor^c^ N(%)	(N) Patients	Diagnosis N(%)	Disease status N(%)	GVHD prophylaxis^d^	Incidence of cGVHD
[15]	2024	R	RIC	MRD 19(52)	35 **rituximab** post-HCT	MCL 13(37.1)	PR 19(54)	CsA + MMF	20 % vs.35 %,p < 0.05
				MUD 13(37)	vs. 43 control	high-risk CLL 22(62.9)	CR 13(37)		(5-y)
[16]	2017	R	MAC	MMRD 30(60)	50 EBV viremia treated with rituximab	AML 18(36)	CR 37(74)	CsA + MMF + MTX	36.1 vs.54.1 %,p = 0.0579
				MUD 18(32)	vs. 52 control	ALL 10(20)	PR/NR 9(18)		(2-y)
[17]	2023	P	RIC	MRD 24(77)	51 **ofatumumab** pre-HSCT	Diffuse large B-cell lymphoma 21(68)	CR 19(61)	SRL + TAC	54 %
				MMRD 7(23)		Mantle cell lymphoma 5(16)	PR 8(26)		(184-d)
[18]	2024	P	MAC	NA	44 AC	Non-Hodgkin Lymphoma 16(36)	Chemo-sensitive 27(61)	SRL + TAC + MTX	4.5 % vs 28.5 %,p = 0.0002
					vs. 39 TMS	CLL 9(20)	Chemo-resistant 14(32)		(severe)
[19]	2020	R	RIC	MUD 115(57.2)	201 **alemtuzumab** T-cell depletion	AML 88(43.8)	CR 131(65.2)	CsA + MTX	4 %/7 %
				MSD 86(42.8)		Non-Hodgkin Lymphoma 38(18.9)	Untreated/chronic phase 25(12.4)		(cGVHD/overlap syndrome)
[20]	2021	R	MAC	MRD 42(51.2)	82 **alemtuzumab** T-cell depletion	FA	Transfusion dependency 100(100)	CsA/TAC	2.44 %
				MUD 23(28.0)					(5-y)
[21]	2024	R	MAC,137(35 %)	MUD	397 **alemtuzumab** T-cell depletion	non-SCID IEI 118(29.7)	NA	CNI/+MMF/MTX	43 % vs 47 %
			RIC,169(42 %)			ALL 104(26.2)			(3-y)
			RTC,91(23 %)						
[22]	2019	R	RIC	MUD 12(57)	21 **BV** pre-HSCT	Hodgkin lymphoma	Chemosensitive 12(57 %)	CsA/+MTX	43 % vs 47 %
				MSD 9(43)	vs. 51 control				(3-y)
[23]	2018	R	RIC 169(78)	MRD 125(60)	210 **BV** pre-HSCT	Hodgkin lymphoma	CR 83(40 %)	NA	41 % vs. 48 %
				MUD 85(40)	vs. 218 control				(3-y)
[24]	2024	P	Flu/Bu, 28 (96)	MUD 17 (59)	29 **tocilizumab** post-HSCT	AML 15(52)	AL-MCR1 10(34)	TAC + MTX + TOC	7.70 %
				MRD 12 (41)					(1-y severe)
[25]	2022	R	Treosulfan,12(60)	MRD	20 **daratumumab** pre-HSCT	Chemorefractory AML	Relapsed refractory 14(70)	Tocilizumab Abataccepept	7.70 %
			TBI,8(9)				Primary refractory 6(28)		(1-y)
[26]	2020	R	Flu/Mel,8(24)	MSD 17(50)	55 **daratumumab** post-HSCT	Standard risk MM 22(65)	VGPR 12(35)	TAC + MTX/MMF + Cy	12 %
			Flu/Mel,5(15)	MUD 11(32)		High risk MM 12(35)	PR 11(32)		(160-d)
			Flu/Cy/TBI,5(15)						
[27]	2022	R	MAC	MRD	239 **basiliximab** post-HSCT	ALL 117(48.95)	CR,MRD-194(81.17)	CsA/TAC + MTX + MMF + ATG	7.7 %/9.8 %/12.3 %

a R:retrospective study; P:prospective study.

b RIC: reduced intensity conditioning; MAC:myeloablative conditioning; Flu:Fludarabine; Bu:Busulfan; TBI:Total Body Irradiation (TBI) Preconditioning; Mel: Melphalan; Cy:Cyclophosphamide.

c Related MUD:Matching Unrelated Donor; MMRD:Mismatch Relative Donor; MRD:Matching Relative Donor; MSD:Matching Sibling Donor;.

d CsA:Cyclosporine A; MMF:mycophenolate mofetil; MTX:Methotrexate; SRL:Sirolimus; TAC:Tacrolimus; CNI:calcineurin inhibitor; ATG:Antithymocyte Globulin.

**Table 2:** The applications of mAbs in treatment cGVHD.

Target	Drug	Study type/Patients Phase	Efficiency			AEs		Ref
			OS/FFS	ORR (CR,PR)	CS-re duction^1^	Nonhematological AEs	Hematological AEs^1^	
B-cells	Anti-CD20 Rituximab	Phase II/72 37 vs. 35 cGVHD	6-m: 14%^b^ vs. 17%	6-m: 27%^c^ vs. 26%	By 50%: 29% (vs. 26%)	Grade 3-5: 29.7% vs. 57.1% Fatal infection: n=2 vs. 2	Grade 3-4 N: 13.5% vs. 5.7% Grade 3-4 L: 2.7% vs. 0	[28]
		Phase II/29 SR-cGVHD	1-y: 96.5%	1-y: 71% (8%, 63%)	By 50%: 57%	SAE: 38% Fatal osteomyelitis n=1	Grade 3 L: 3.4% (1/29)	[29]
		Phase II/25 cGVHD	2-y: 82%/1-y: 80% 2-y: 67%	1-y: 88% (84%, 4%)	By 100%: 80%	Hypogammaglobulinemia: 68%; CMV reactivation: 22%; Fungal infection: 12%	NA	[30]
		Phase II/24 cGVHD	1-y: 83%	1-y: 83% (29%, 54%	By 100%: 74%	SAE: 45.8% (11/24) Infection n=7 Fatal PML n=1	Grade 3-4 L: 50% Grade 3-4 N: 21%	[14]
		Retrospective/29 cGVHD	1-y: 72%/1-y: 24%	3-m: 31% (7%, 24%)	median dose: (mg/kg) 0.42 → 0.15 at 3 m	Infection: 24% Fatal: 13.8%		[31]
		Retrospective/13 cGVHD	1-y: 69%	54%	median dose: (mg/d) 27 → 11 at 1 year	0 IRR^1^s	NA	[32]
		Retrospective/69 cGVHD	1-y: 87% /1-y: 75%	1-y:41% CI of resolution	By 100%: 39% (27/69)	Infection: 50% (of NRM)	NA	[33]
	Anti-CD20 Ofatumumab	Phase I/12 cGVHD	1-y: 82%^	1-y: 80% (36%, 44%)	median dose: (mg/kg) 0.4 → 0.07 at 6 m	AE: 14% (4/29) (no DLT)	NA	[34]
		Phase II/38 cGVHD	/1-y: 53%	6-m: 62.5% (9.4%, 53.1%)	median dose: (mg/kg) 1→ 0.14 at 6 m	Infusion-related: 44.7% Viral infection: 68.4%	Grade 3 A: 2.6%, Grade 3 N: 2.6% Grade 4 N: 2.6%	[35]
T-cells	Anti-CD52 Alemtuzumab	Phase I/13 SR-cGVHD	2-y: 69.2% 3-y: 59.3%/1-y: 30.8%, 2-y: 23.1%	3-m: 70% (30%, 40%)	61.6%	Infection: 75% (at dose level 3) Grade 3 TMA^1^: 7.7% (at dose level 3)	Grade 4 N+T: 15% (2/13) Grade 3 N: 7.7% (1/13)	[36]
	Anti-CD30 Brentuximab	Phase I/17 SR-cGVHD	NA	6-m: 47% (0, 47%)	By 50%: 65% By 100%: 17.4%	Peripheral neuropathy: 41%	NA	[37]
	Anti-IL-6 Tocilizumab	Retrospective/11 SR-cGVHD	2-y: 82%	1-y: 70% (0, 70%)	median dose: (mg/kg) 0.16→ 0.06 at 12 m	Infection: 18.2% (2/11)	G+ T: 18.2% (2/11)	[38]
		Retrospective/5 cGVHD	NA	3-m: 100%^d^	By 100%: 20%	Infection: 60%		[39]
Macrophages	Anti-CSF1 Axatilimab	Phase I/II/40 SR-cGVHD 17/13	/1-y: 77% (Ph II)	3-m: 82%(Ph II)	22%	Grade 3-4 AE: 20% (8/40) Infection: 48%	Grade 2 N: 1/40 Grade 1 T: 1/40 Grade 3 T: 1/40	[40]

b 6-m treatment success (SCR at 6 months without crossover, recurrent malignancy or death): 14%

c 6-m SCR (significant clinical response): 26%

d subjective improvement reported by patients at 3 months

1 Approximate representation: CS-reduction: corticosteroid dose reduction;Hematological AE: N: Neutropenia; L: Lymphopenia; A: Anemia T: Thrombocytopenia ; G: Granulocytopenia; IRR: infusion-related reaction;TMA:thrombotic microangiopathy

**Table 3:** Current clinical applications of mAbs in cGVHD.

Medication Phase	Target/Drug
Prophylaxis	Anti-CD20/Rituximab,ofatumumab
	Anti-IL-6R/Tocilizumab
	Anti-NKG2A/Monalizumab
	Anti-CD52/Alemtuzumab
	Anti-CD38/Daratumumab
	Anti-CD25/Basiliximab
Treatment	Anti-CD20/Rituximab
	Anti-CD20/Ofatumumab
	Anti-CD52/Alemtuzumab
	Anti-CD30/Brentuximab
	Anti-IL-6R/Tocilizumab
	Anti-TNFα/Infliximab
	Anti-CSF/Axatilimab

## 2 Targeting B cells

### 2.1 Anti-CD20 mAbs (rituximab, ofatumumab and obinutuzumab)

CD20 is a cell surface marker that is exclusively expressed on pre-B lymphocytes and mature B lymphocytes. Anti-CD20 mAbs can inhibit the growth of B-cell clones in vivo and deplete B cells [41]. Rituximab is a chimeric mAb that targets CD20. The binding of rituximab to CD20 can destroy lymphocytes through various mechanisms, including antibody-dependent cell-mediated cytotoxicity, complement-dependent cytotoxicity (CDC), and direct apoptosis [42]. Like rituximab, ofatumumab is another fully human anti-CD20 mAb that targets and destroys B lymphocytes [43]. The unique binding site of ofatumumab and an increase in the induction efficacy of CDC may be necessary for overcoming rituximab resistance [44]. In 2-mouse cGVHD models, rituximab can prevent the induction of autoimmune-like cGVHD and maintain the role of GVL before signs of cGVHD appear [45].

In 2 prospective clinical trials, rituximab prophylaxis successfully decreased rates of cGVHD [46,47]. The results of the above studies revealed that the cumulative incidence of cGVHD was 20 %, and the incidence of cGVHD requiring steroids was significantly reduced to 31 %. The patients in the above studies were included in a retrospective analysis: 35 patients given prophylactic rituximab infused at 375 mg/m2 weekly for 4 weeks on days 56, 63, 70, and 77 post-HSCT had an 8-year cumulative incidence of cGVHD of 20.0 % and did not develop late-stage cGVHD in the 14 months after HSCT. Furthermore, the proportions of IgD+/CD38+ transitional B cells and antibodies to HY in female-to-male patients decreased over a 10-year follow-up period [15]. Rituximab was shown to be effective and feasible for preventing cGVHD after HCT, and the effects on donor immune reestablishment and function were long-lasting. In addition, a retrospective study revealed that the cumulative incidence of cGVHD in the rituximab group was lower than that in the control group when patients with Epstein–Barr virus (EBV) viremia occurring within 100 days of HSCT were given rituximab (p = 0.0579), and in multifactorial analysis, rituximab was an independent factor for the reduction in the cumulative incidence of cGVHD (p = 0.0069) [16]. Obinutuzumab is a novel potent human anti-CD20 mAb. cGVHD prophylaxis with obinutuzumab administered (1000 mg) at 3, 6, 9 and 12 months after HSCT was investigated in a randomized placebo-controlled trial (NCT02867384), which revealed a significantly reduced rate of steroid-refractory cGVHD at 1 year post-hematopoietic cell transplantation (HCT) (11 % vs. 38 %, p = 0.008), and relapse-free survival (CRFS) was greater in the obinutuzumab group (43 % vs. 30 %, p = 0.009) [48]. The safety and efficacy of ofatumumab-based reduced-intensity conditioning (RIC) in allogeneic transplantation (allo-SCT) for high-risk non-Hodgkin's lymphoma (NHL) were validated: the incidence of the primary endpoint, grade 3–4 acute GVHD (aGVHD), was 16 %, and cGVHD was observed in 54 % of the patients; the anti-tumor and anti-GVHD effects of ofatumumab in additional cohort studies are pending [17]. These studies provide new insights into the prevention of GVHD and indicate the need for further expansion for broad population validation.

Rituximab as initial CS-free treatment for extensive cGVHD was evaluated in phase II trials; 88 % of the patients responded, and the median time for 77 % of the respondents to successfully withdraw from all IST treatments was 300 days. However, 37 % of the patients experienced cGVHD recurrence within two years, and immunosuppressant drugs needed to be restored [30]. In a follow-up retrospective evaluation of 69 patients (25 of whom initially participated in the phase II trial mentioned above), further evidence of the effectiveness of the method was published: the cumulative incidences of cGVHD resolution with rituximab at 1 year, 2 years, and 3 years were 41 %, 69 %, and 77 %, respectively. The median follow-up for surviving patients was 47 months; no systemic CSs were used in 27 patients, and 67 % of these patients received ≤10 mg/kg [33]. Rituximab is also expected to stabilize or improve lung function in patients with bronchiolitis obliterans syndrome (BOS) after HSCT: the forced expiratory volume in 1 s (FEV1) increased in 54 % (7/13) of patients, and the mean daily dose of prednisone decreased from 27 mg to 11 mg at 12 months after treatment [32]. The efficacy and safety of rituximab were evaluated in 29 patients with cGVHD at the Regensburg University Transplantation Center. The overall response rate was 31 % at three months, with higher response rates in patients with steroid-dependent cGVHD. Notably, 7 patients (24 %) had severe infection, and unresponsive patients seemed to be more prone to infectious complications [31]. Arai, S. et al. published the results of a randomized phase II crossover study of imatinib or rituximab in the treatment of patients with sclerosing cGVHD, of which 10 of the 37 patients (27 %) in the rituximab group experienced a significant clinical response (SCR), and more activated B cells [CD27(+)] were observed in the rituximab group (p = 0.01) [28]. A phase II prospective multicenter trial tested the efficacy of rituximab combined with CSs and cyclosporine A (CsA) as a first-line treatment for newly diagnosed cGVHD. The results revealed that 20 of 24 patients (83 %) responded within one year [14]. These studies provide insights into adding rituximab to standard first-line therapy to improve the long-term control of cGVHD. An evaluation of sequential therapy with rituximab and nilotinib in 29 patients with steroid-refractory sclerosing cGVHD revealed more profound and longer-lasting objective responses, at 71 %, and a higher survival rate (1 year, 96.6 %). In most responders, the CSs dose was reduced to less than 50 % [29]. In addition, the infiltration of B lymphocytes (CD20^+^ cells) in muscle is associated with an abnormally high number of CD20^+^ cells in the peripheral blood. When high-dose methylprednisolone in combination with rituximab was used, the first case of pediatric cGVHD-associated polymyositis that was successfully treated with rituximab was reported [49]. In summary, the combination of rituximab and existing first-line therapies can achieve a relatively high effective rate and can reduce patient dependence on steroids and accelerate steroid withdrawal. In addition, rituximab can improve the outcomes of patients with prolonged disease in specific organs, such as the lungs and skin muscles, to a certain extent.

A phase I clinical trial revealed that dose-limiting toxicity (DLT) was not observed when ofatumumab was administered intravenously at three escalating dose levels (300 mg, 700 mg, and 1000 mg) on days 1 and 14 of initial glucocorticoid therapy [34]. A recent one-arm phase II trial revealed that ofatumumab was safe and effective as an initial treatment for cGVHD: the 6-month clinician-reported and 2014 NIH-defined overall response rate was 62.5 %, and the 12-month failure-free survival (FFS) was 53.1 % [35]. Ofatumumab may be a milder anti-CD20 mAb used to control cGVHD, which needs to be further expanded and verified.

### 2.2 Anti-BAFF mAb (belimumab)

Elevated BAFF levels promote the survival of activated autoreactive and alloreactive B cells by increasing the B-cell receptor (BCR) response and intracellular Syk and BLNK phosphorylation [50,51]. In addition, BAFF increased the expression of NOTCH2 on B cells and promoted the reactivity of BCRS to substitute antigens and NOTCH ligands [52]. Wei Jia et al. reported that cGVHD manifestations in mice were associated with high BAFF/B-cell ratios and the persistence of BCR-activated B cells in peripheral blood and lesional tissue [52]. Further studies have shown that when belimumab blocks BAFF signaling, preventing cGVHD by inhibiting B-cell activation in patients with active chronic GVHD is feasible [53]. Belimumab is a recombinant immunoglobulin G1-λ human mAb that functions by blocking the interaction of soluble BAFF with BAFF-R, TACI, and BCMA [54].

Preliminary data from a single-center phase 1 trial revealed for the first time that prevention of cGVHD is possible with belimumab and that this drug is well tolerated. Belimumab was administered intravenously (i.v.) at 10 mg/kg every 2 weeks for 3 doses followed by 4 doses at monthly intervals, for a total of 7 doses, starting 50–80 days after allo-HCT; data are being collected [55]. Studies have shown that BAFF levels rise after rituximab is administered [56] and that increased BAFF levels persist after initial B-cell regeneration following treatment with B-cell depletion [57]. Blocking the effect of high serum BAFF levels may have a favorable effect on the reconstruction of B cells after consumption, and the synergistic effects of this combination have been reported in case reports [58].

### 2.3 Anti-CD38 mAb (daratumumab)

CD38 is a type II transmembrane glycoprotein that was first identified as a marker of T lymphocyte activation [59]. By disrupting the proportion of functional subsets of T cells and impeding T-cell activation and migration, daratumumab prevents experimental GVHD [60]. Daratumumab participates in immune-mediated antibody-dependent cytotoxicity, antibody-dependent cellular phagocytosis and component-dependent cytotoxicity through Fc-dependent immune effector mechanisms, thereby reducing the number of CD38-positive immunosuppressive cells, including T regulatory cells (Tregs), natural killer (NK) cells, regulatory B cells, and myeloid-derived suppressor cells, affecting CD38 nonmyeloid tumor cells that mediate GVHD and theoretically affects GVHD episodes [61,62].

The use of daratumumab-based therapy after allo-HCT is not well described, with a theoretical concern that its use may cause a flare-up of GVHD in part owing to its effects on the T-cell milieu. The combined addition of venetoclax and daratumumab to a transplant prep regimen for children with refractory acute myeloid leukemia resulted in a cumulative incidence of cGVHD of 7.7 % and an event-free survival rate of 44 % [25]. Retrospective studies evaluating the safety and efficacy of daratumumab after HCT revealed a cumulative incidence of cGVHD of 4 % (0–7%) within two years of initiation of daratumumab, of which 3 % had extensive cGVHD, and the study suggested no significant effect on the induction of cGVHD and synergism with existing cGVHD [63]. The incidence of GVHD in 34 patients with recurrent multiple myeloma (MM) treated with daratumumab was evaluated in a multicenter retrospective study, and the cumulative incidence of GVHD at 100 and 160 days after starting daratumumab was 6 % and 12 %, respectively [26]. The timing of the use of daratumumab requires further study and does not appear to have an adverse effect (AE) on cGVHD.

In addition, a case of pediatric oral GVHD ameliorated and progressing to nephrotic syndrome and then end-stage renal disease traeted with daratumumabwas previously reported: the biopsy was suggestive of membranous nephropathy (MN). The patient had steroid toxicity and 0 % CD19 cells in lymphocyte subsets according to flow cytometry, but treatment with 2 doses of daratumumab resulted in remission of nephrotic syndrome, and steroids and tacrolimus were discontinued after the 3rd dose, suggesting that plasma cell depletion therapy may be an important novel option [64].

## 3 Targeting T-cells

### 3.1 Anti-CD52 mAb (alemtuzumab)

CD52 is a novel costimulatory molecule that induces CD4^+^ regulatory T cells in GVHD [65]. Alemtuzumab, alternatively referred to as Campaign-1H, is an mAb of humanized IgG1 designed to bind to human CD52.

A retrospective study demonstrated that grafting, the size of which was determined by the T-cell content of peripheral blood stem cell grafts, was safe and led to early T-cell immune reconstitution in patients with nonmalignant disease treated with alemtuzumab serum, and cGVHD was not observed in a cohort of 26 patients (median follow-up of 899 days) [66]. Early studies employed high doses of alemtuzumab (a total dose of 100 mg) [67,68]. However, in the current treatment landscape, safe de-escalation to total doses of 30–60 mg is achievable without compromising clinical outcomes [69,70]. In a study comparing three different doses of alemtuzumab for the prevention of GVHD, the incidence of acute grade I-IV GVHD in 313 patients was 74 %, 65 %, and 64 %, and the incidence of chronic GHVD was 36 %, 32 %, and 41 % when this treatment was administered at 100 mg, 60 mg, or 50 mg, respectively [71]. Recent research has shown that when high-dose alemtuzumab/cyclosporine was used as the observation arm in RIC allo-HSCT, the primary endpoint was the cumulative incidence of severe cGVHD, which was lower than that of tacrolimus/methotrexate/silomoxetine, with lower 1- and 5-year incidences of severe cGVHD (0 % vs. 10.3 %, 4.5 % vs. 28.5 %, overall p = 0.0002), as well as a lower incidence of any grade (p = 0.003) and moderately severe (p < 0.0001) cGVHD, as well, with no difference in 5-year overall survival (OS) or NRM, preliminarily suggesting that an alemtuzumab-based regimen successfully reduces the incidence and severity of cGVHD after RIC allo-HSCT [18]. Other data from 201 adult patients who received alemtuzumab and RIC allo-HSCT revealed that the cumulative incidence of aGVHD grades II-IV (III-IV) was 20 % (8 %), and the cumulative incidence of cGVHD and overlap syndrome was 4 % and 7 %, respectively [19]. In addition, the 5-year cGVHD event-free survival rate after transplantation in 82 patients with Fanconi anemia receiving a fludarabine-cyclophosphamide (Flu-Cy)-based conditioning regimen and in vivo T-cell depletion with alemtuzumab was 75–4% [20]. Additional studies have identified the combination of in vivo T-cell depletion via alemtuzumab and dual GVHD prophylaxis (calcineurin inhibitor and mycophenolate mofetil) as a potential strategy to reduce the risk of severe GVHD in pediatric peripheral blood stem cell (PBSC) recipients. A total of 397 children underwent initial matching unrelated donor(MUD)-HSCT with a median follow-up time of 3.1 years, with a lower incidence of cGVHD in the PBSC transplantation group than in the bone marrow transplantation group (6 % vs. 11 %; p = 0.03) and a CD3^+^ T-cell dose >5 × 10^8^/kg; the use of PBSCs was an independent predictor of grade II-IV aGVHD [21]. Alemtuzumab-based conditioning regimens have been shown to reduce the risk of cGVHD, according to previous studies. Especially in SAA patients, alemtuzumab is preferred for replacing the anti-thymocyte globulin (ATG) conditioning regimen and preventing GVHD [72,73]. In only one study on the treatment of cGVHD, the 12-week response rate to alenzumab in 13SR-cGVHD patients was 70 % [36].

### 3.2 Anti-CD30 mAb (brentuximab)

CD30 is expressed by activated CD4^+^ and CD8^+^ T cells and Tregs, as well as some B cells, and is involved in the development of GVHD [74].

Brentuximab vedotin (BV) is a CD30-targeted antibody‒drug conjugate. Antibody‒drug coupled BV and programmed cell death protein-1 (PD-1) blockers targeting CD30 showed durable clinical activity in patients with relapsed/refractory 10.13039/100023110HL (r/r) who underwent HSCT, and the case data support that PD-1 blockade in combination with BV promotes the antilymphoma effect of grafts after allo-HSCT but may be associated with GVHD development [75]. In addition, some studies have shown that the use of anti-PD-1 mAbs prior to allo-HSCT may increase the risk of GVHD [76] because PD-1 inhibition is associated with the expansion and activation of preexisting T cells [77]. In a retrospective multicenter study of 21 BV-pretreated patients and 51 controls, the 3-year cumulative incidence of cGVHD was 43 % in the BV group and 47 % in the non-BV group, with no significant difference [22]. However, in another study comparing the results of 210 patients treated with BV prior to allogeneic SCT with 218 patients who did not receive BV, with a median follow-up of 41 months, BV significantly reduced the risk of cGVHD [risk ratio = 0.64; 95 % confidence interval (CI) = 0.45–0.92; p < 0.02] [23]. The above studies show no clear warning signs of GVHD risk when BV is used before or after allo-HSCT and suggest better clinical outcomes when BV is used before transplantation.

Two studies thus far have evaluated the efficacy of BV as a treatment option for GVHD. The drug achieved an ORR of 38.2 % in a phase 1 multicenter trial of 34 patients with SR-aGVHD (on day 28), and 1 case of DLT (sepsis) was observed [78]. A phase I trial for SR-cGVHD resulted in a 47 % ORR, and 41 % of patients experienced grade 3 or 4 AEs (especially severe peripheral neuropathy), indicating that further treatment modifications may be required for toxicity management [37]. This treatment appears to be poorly tolerated in patients with cGVHD, so more extensive studies are needed.

### 3.3 Anti-IL-6R mAb (tocilizumab)

IL-6 promotes donor B-cell-derived IgG-induced Th17 cell infiltration and germinal center formation, thus contributing to the pathogenesis of GVHD [79,80]. IL-6 has various downstream effects (through STAT3 and MAPK signaling) and plays a vital role in initiating the GVHD inflammatory response [81]. In addition, IL-6 induces the differentiation of helper T cells (especially those producing IL-17), which are known to be involved in the inflammatory state [82]. In a humanized mouse model, an anti-IL-6 mAb combined with cyclophosphamine substantially reduced liver and lung damage after transplantation, delayed the occurrence of cGVHD, reduced the number of NK cells, and increased the number of Tregs [83]. Tocilizumab is a humanized IgG1 IL-6 receptor antibody that has been approved for treating various inflammatory diseases, including rheumatoid arthritis and release syndrome, after CAR-T-cell infusion [84].

A single-center retrospective study revealed that treatment of CRS with or without tocilizumab in a PTCy-based haplo-HCT population resulted in a higher cumulative incidence of cGVHD at 1 year in the observation group than in the control group (64 % vs. 24 %; p = 0.05) [85]. The impact of tocilizumab treatment for CRS on GVHD needs to be evaluated in larger prospective studies In another phase I study, patients received busulfan-based myeloablative modulation, and GVHD prophylaxis consisted of tacrolimus and methotrexate plus tocilizumab administered on days 1 and 100; the cumulative incidence of overall cGVHD [using the National Institutes of Health (NIH) consensus criteria] was 64.8 % (95 % CI: 42.4–80.3), with a 12-month incidence of severe cGVHD of 7.7 % (95 % CI: 1.3–22.1); the 1-year GRFS was 37.9 % (95 % CI: 20.954.9), which was significantly greater than the predetermined historical probability of 20 % (p = 0.023) and was accompanied by preservation of microbial diversity and attenuation of enterococcal dominance [24].

This retrospective study revealed that tocilizumab was safe and effective in treating severe cGVHD (n = 11): 7/10 patients (70 %) achieved partial remission [38]. A phase II clinical trial of cGVHD with ineffective multiline therapy is underway (NCT02174263). Five pediatric patients with cGVHD were unresponsive or intolerant to multiple prior treatment regimens, and all patients reported subjective improvement after tocilizumab infusion, including 80 % of patients with sclerotic disease [39]. Tocilizumab combined with an antiviral drug for the treatment of severe COVID-19 with extensive chronic GVHD (involving the liver, lungs, eyes, skin, and recurrent lung infections) was reported, and the patient recovered and was discharged from the hospital on day 31, with no worsening of pulmonary GVHD or signs of pulmonary fibrosis at the 6-month follow-up; the analysis revealed that tocilizumab may have improved the number of NK cell counts after day 10, and its inhibition of the IL6 axis may indirectly promote NK cell function [86]. Thus, tocilizumab has an initial clinical advantage in improving the skin and preventing pulmonary fibrotic progression. On the basis of the safety tests, the drug needs to be expanded into clinical trials in the cGVHD population in which multiline therapy has failed.

Tocilizumab has also been studied for the control of aGVHD. In a phase 3 randomized, double-blind trial, tocilizumab did not significantly decrease the incidence of grade 2–4 aGVHD in HLA-matched voluntary unrelated donor (VUD) recipients: the incidence of grade 2–4 aGVHD on day 100 was 36 % in the placebo group compared with 27 % in the tocilizumab group (p = 0.23) [87]. Data from a retrospective study revealed that 10 of 16 patients with SR-aGVHD [62.5 %; 95 % Cl (0.39–82)] achieved complete remission within a median of 11 days (range, 2–28 days) after the initiation of treatment with tocilizumab, which appears to be a highly active agent for the treatment of severe SR-lower-GI aGVHD [88]. In addition, research shows that intrathecal tocilizumab has been reported to be safe and effective in the treatment of central nervous system(CNS) aGVHD with predominantly elevated IL-6, with patients treated with effective seizure control and significant reductions in both peripheral blood and cerebrospinal fluid IL-6 levels [89].

### 3.4 Anti-TNF-α mAb (infliximab)

TNF-α initiates the proliferation of Tregs in vivo while limiting the expansion of CD4^+^ and CD8^+^ conventional T cells (Tcons) to induce GVHD [90]. Infliximab contains a constant sequence of human IgG1 and a variable region of mouse IgG and effectively blocks the binding of TNF-α to its soluble and membrane receptors [91].

There are few clinical studies of this drug with cGVHD. In one case report, a patient with refractory Hodgkin's lymphoma who underwent CBT for 1 year complained of intermittent abdominal pain and bloody diarrhea, and colonoscopy revealed multiple longitudinal colonic ulcers with a cobblestone appearance, which was considered a Crohn's disease (CD)-like manifestation of GVHD in the gastrointestinal tract. The patient's abdominal symptoms improved rapidly after the administration of anti-TNF alpha mAb, infliximab, and adalimumab [92]. Another patient with refractory anal fistula 1 year after transplantation for t(8; 21)-positive acute myeloid leukemia presented with noncaseating epithelioid granuloma on examination, and the abdominal symptoms and anal fistula rapidly improved after infliximab treatment [93].

### 3.5 Anti-IL-4/-13R mAb (dupilumab)

Investigators further reported that patients' elevated levels of IgE, Th2 cells, regulatory T cells, and eosinophils may play important roles in type 2 inflammation in atopic dermatitis (AD)-like GVHD, indicating that blocking IL-4 and IL-13 is a promising therapeutic prospect, significantly improving patients' nodular itchy rash compared with other medications (which may be associated with neurogenic involvement of the skin) [94].

The first case of the use of dupilumab for the treatment of AD-like GVHD was reported: a clinical improvement in GVHD and a reduction in pruritus in 3/4 (75 %) of the patients, as well as an effective reduction in the duration and amount of systemic immunosuppression in the pediatric transplant population, resulting in an improvement in the QoL of the patients [95]. Similar cases have been reported with the following results: a significant reduction in pruritus within a few days of the first 300 mg dose of dupilumab in patients with AD-like GVHD; sustained effectiveness after the subcutaneous administration of 200 mg of dupilumab every two weeks; withdrawal of steroids from treatment within 3 months of administration; and significant improvement in the patient's mood, sleep, and QoL [96].

### 3.6 Anti-CD25 mAb (basiliximab)

IL-2/IL-2Rα signaling controls the proliferation and activation of T cells. Basiliximab is a human/mouse chimeric mAb that targets the interleukin-2 receptor α chain (IL-2Ra) or CD25 on activated T lymphocytes. Blocking this subunit prevents heterologous trimerization and the IL-2Ra chain from participating in the IL-2R complex, thereby preventing rapid clonal expansion of subsequently activated T lymphocytes [97,98]. Given that basiliximab can selectively eliminate only donor-specific alloreactive T cells without affecting the resting T cells present in the graft, basiliximab can prevent GVHD without affecting immune function [99].

In patients with high-risk acute leukemia, the use of an idarubicin (IDA)-intensive conditioning regimen combined with ATG and basiliximab can prevent GVHD; the cumulative incidences of limited and extensive cGVHD are 19.4 % and 13.8 %, respectively [100]. A retrospective study analyzed the results of 239 patients receiving haplo-HSCT combined with basiliximab and different doses of ATG for GVHD prevention: the 2-year cumulative incidence of total cGVHD and extensive cGVHD was 9.8 ± 2.2 % and 4.1 ± 1.5 %, respectively, and compared with high-dose ATG (>6 mg/kg), low-dose ATG (<6 mg/kg) significantly reduced the risk of CMV viremia (52.38 % vs. 79.35 %, p < 0.001), and low-dose ATG in combination with basiliximab seems to be more suitable for balancing infection control and GVHD prevention [27].

Basiliximab seems more prominent in aGVHD control study. Data from a prospective multicenter clinical trial of 65 patients with severe SR-aGVHD from 6 centers revealed that the combination of basiliximab and etanercept resulted in overall remission [complete remission and partial remission (CR + PR)] of 90.8 %, which was significantly superior to the 2-year OS rate (54.7 % versus 14.8 %, p < 0.001) [101]. Another large-scale follow-up study revealed that the cumulative incidence of 56-day overall remission with basiliximab for patients with SR-aGVHD was 80.1 % and that the cumulative 4-year post-treatment incidences of total and severe cGVHD were 44.8 % and 2.2 %, respectively [102].

### 3.7 Anti-IL-12/-23R mAbs

IL-12 plays a crucial role in the differentiation of CD4^+^ T cells into Th1 cells, and IL-23 stabilizes the Th17 phenotype; these cytokines have a common p40 subunit [[103], [104], [105]]. Anti-p40 mAbs attenuate experimental cGVHD by inhibiting IFN-γ/IL-17-producing cells and thus may be a promising therapeutic strategy for the prevention and treatment of cGVHD [106].

In addition, the metabolite retinoic acid (RA) is known to promote Gl-GVHD in mice via allogeneic reactive T cells expressing RA receptor-α (RARα), and studies using a targeting candidate protein approach have predicted the phenotype of RA-responsive T cells in the context of increased microenvironmental interleukin-23 (IL-23) and confirmed the presence of a RARahi CD8 T-cell population via serial immunostaining and coexpression of the effector T-cell transcription factor T-bet and IL-23-specific receptor (IL-23R), which targets IL-23-rich conditions and represents a potential new therapeutic target [107]. Pidala J et al. provided the first evidence that IL-12/IL-23p40 neutralization can polarize donor-versus-host allogeneic responses in vivo; however, there was no significant difference in the cumulative incidence of any grade of cGVHD between groups [108].

### 3.8 Anti-INF-γ mAbs

Type II interferons (IFN-γ) play intricate roles in cGVHD. Akimasa Saito et al. revealed that IFN-γ promotes TGFb1 production by apoptotic keratinocytes (KCs), thus mediating the development of widespread sclerodermatous changes in KC-targeting GVHD [109]. Scholars have used a mAb to block the colony-stimulating factor 1 receptor (CSF1R) to explore its effects on a CNS model of cGVHD and reported that when IFN-γ receptor-deficient grafts were used in the model system, reduced expression of MHC class II on bone marrow-derived macrophages (BMDMs) was observed, and the animals did not develop neuroinflammation, reaffirming the possibility of disrupting this pathway via an anticytokine strategy (emapalumab blockade of IFN-γ) to disrupt the possible opportunities of this pathway [110]. However, another study revealed that IFN-γ R/STAT1 signaling in antigen-presenting cells functions as an immune rheostat to restrain autoinflammation by suppressing endogenous antigen presentation under inflammatory conditions while increasing responses against exogenous antigens [111]. In addition, the data from the largest HSCT cohort using emapalumab show that it can prevent or treat people at high risk of implantation failure, and studies are ongoing [112]. The effect of the absence of IFN-γ receptor signaling in donor cells on GVHD remains to be further explored and determined.

### 3.9 Anti-CD45 mAbs

CD45 is highly expressed in the hematopoietic system and is involved in lymphocyte activation and maturation, as well as thymic selection [113]. CD45^−^antibody‒drug conjugates (ADCs) effectively protect against cGVHD. Asim Saha et al. reported that CD45-ADCs can effectively deplete host HSPCs and lymphoid cells and allow robust donor engraftment and lymphohematopoietic recovery without a propensity for GVHD in preclinical mouse models [114]. Similarly, Meera A. Srikanthan et al. reported that CD45-ADC conditioning resulted in HSC depletion and helped donor engraftment comparable with cyclophosphamide treatment but with less toxicity [115].

### 3.10 Anti-OX40 mAbs

Using RNA sequencing with RT‒PCR and immunohistochemistry validation, investigators verified that sclerosing cutaneous cGVHD may have strong Th1-associated upregulation and a distinctive TSLP-OX40 signature, which provides ideas for future targeted therapies [116].

## 4 Targeting macrophages

### 4.1 Anti-CSF-1R mAb (axatilimab)

CSF-1 is involved in the production, differentiation, and function of macrophages and, together with allogeneic antibody deposition, induces a transforming growth factor β-high environment in target tissues, leading to scleroderma and BOS [117]. Anti-CSF-1R-treated mice retain donor BMDMs, activated microglia, and CD8^+^ and CD4^+^ T cells and express local cytokines in the brain, which supports the hypothesis that CSF-1R antibody blockade may also be a useful strategy to prevent/treat 10.13039/100015862CNS cGVHD [118]. Axatilimab is a high-affinity (KD 4–8 pm) humanized IgG4 mAb that can block the binding of CSF-1 and interleukin-34 ligands and preferentially removes nonclassic monocytes in peripheral blood. Its safety is consistent with its mechanism of action [119].

In a recently published phase I/II study, the safety and efficacy of this mAb were further verified. In a phase I study, a biological dose of 3 mg/kg was administered every 4 weeks to obtain the best response, whereas in a phase II study, the ORR of the primary endpoint on the first day of the 7th cycle was 50 %, the ORR was 82 % in the first 6 cycles, and 58 % of patients reported significant improvements in clinical outcomes [40]. The latest data suggest that axatilimab at 0.3 mg/kg Q2W resulted in the highest response rate and most manageable safety profile compared with 2 higher doses in patients with refractory/recurrent cGVHD; the primary endpoint was the ORR in the first 6 cycles, with approximately 3/4 of patients experiencing a response and a median time to a first response of 1.5 months (range 0.9–5.1). Notably, the responses were observed in fibrosis-dominated organs, including joints and fascia (76 %), lungs (47 %), and skin (27 %), and 55 % of patients reported clinically meaningful changes of ≥7-point improvement in the mLSS. Responses were observed in fibrosis-dominated organs, including joints and fascia (76 %), lungs (47 %), and skin (27 %). This phase II, open-label, randomized study (AGAVE-201) indicates that axatilimab is a new treatment option [120].

### 4.2 Anti-CX3CL1 mAbs

CX3CL1, a chemokine expressed on endothelial cells, can attract mainly monocytes, macrophages and other cells, including NK cells and T cells, which leads to tissue-specific inflammation and fibrosis [121,122]. Preclinical studies have shown that intraperitoneal injection of an anti-CX3cl1 mAb inhibits the development of skin and lung fibrosis in Scl-cGVHD models without any significant AEs, and RNA sequencing analysis of fibrotic skin has shown that mAb treatment significantly decreases the cGVHD-dependent induction of macrophage-associated inflammation and fibrosis-associated gene sets [123].

## 5 Targeting NK cells

### 5.1 Anti-NKG2A mAb (monalizumab)

After allo-HSCT, the quantitative and qualitative reconstruction of NKG2A^+^ NK cells is associated with GVHD [124].

The safety of monalizumab as a maintenance therapy after allo-HSCT was evaluated. At a dose of 1 mg/kg, receptor saturation analysis (RSA) revealed that monalizumab continuously binds to peripheral blood NK cells and can be used safely without reaching the maximum tolerated dose (MTD) [125].

Inhibition of NKG2A alleviates NK cell-mediated graft-versus-tumor effects without triggering GVHD. A prospective study evaluating EBV reactivation after CTLA4Ig-based haploidentical transplantation and its effect on GVHD revealed that the overall prevalence of EBV reactivation was 19 % in 71 patients, and a significant increase in chronic GVHD was observed after EBV reactivation (62.5 % vs. 8 %; p = 0.01), with a significant increase in and persistence of the NKG2A subpopulation of CD56 NK cells, whereas the NKG2C subpopulation decreased [126]. This study may further promote the clinical application of anti-NKG2A mAbs in GVHD, especially in the context of EBV infection.

### 5.2 Potential drugs

Both cGVHD and autoimmune diseases are diseases that result from abnormalities in the immune system. Specifically, cGVHD is caused by transplanted donor immune cells attacking the recipient's tissues, whereas autoimmune diseases are caused by the body's immune system attacking its own tissues. These two types of diseases share many similarities in terms of immune system activation, the release of inflammatory mediators, and tissue damage. Some mAb drugs used in autoimmune diseases can be considered potential drugs that can be developed in the future for combating cGVHD, as they target similar or identical sites in cGVHD and share the same mechanism of action as the drug.

### 5.3 Anti-TNF-α mAbs (adalimumab, golimumab and infliximab)

In the propensity score-matched cohorts of 147 adalimumab-treated and 147 infliximab-treated children in the Real World Study, the 1-year steroid-free clinical remission (SFCR) rates were 63 % and 59 %, respectively [adalimumab odds ratio (OR) 1.4, 95 % CI 0.9–2.4], and adalimumab -treated children were less likely to receive treatment intensification [21 (14 %)] (p < 0.0001). In addition, for both adalimumab and infliximab, the first antitumor necrosis factor drug, children with CD had favorable outcomes at 1 year [127]. Interestingly, Zymfentra is the first FDA-approved subcutaneous formulation of infliximab for inflammatory bowel disease (IBD), which is based on maintenance therapy following intravenous administration.

Adalimumab has also been shown to be effective for up to 5 years in pediatric patients with moderately severe active CD who failed conventional therapy and previous antitumor necrosis factor therapy, and adalimumab has been shown to significantly improve the linear growth rate and QoL of children [128].

### 5.4 Anti-IL-12 mAb (ustekinumab)

Cumulative safety data for ustekinumab in patients with CD for more than 5 years and in patients with ulcerative colitis (UC) for more than 4 years revealed that the incidence of safety events [including AEs, serious AEs (SAEs), infections, and serious infections] was not greater than that in the placebo group [129]. A total of 961 patients with moderate to severe UC were randomly assigned to receive intravenous induction with ustekinumab, and the results revealed that the percentage of those in clinical remission was significantly greater at week 44 (43.8 %, p < 0.01) [130]. In addition, ustekinumab is considered safe and effective for the treatment of pediatric psoriasis; compared with the 5.4 % in the placebo group (p < 0.001), the standard ustekinumab dose provided a response comparable to that of adults at 1 year without unexpected AEs [131].

### 5.5 Anti-IL-23 mAb (guselkumab)

Guselkumab is safe and effective in treating moderately to severely active UC. QUASAR phase 2b data revealed that guselkumab resulted in a higher rate of clinical remission at week 12 (27.6 %) than did the placebo (p < 0.001) [132][], and QUASAR phase 3 data revealed similar results (22.6 %, p < 0.001) [133]. In addition, the use of combination monotherapy for the control of UC has been explored and recommended: patients with the combination therapy with guselkumab and golimumab group experienced a greater clinical response (83 %, p < 0.001) than did those in the golimumab monotherapy group [134]. This combination therapy clinical trial was also conducted in China (CTR20222703).

### 5.6 Anti-BAFF mAb (belimumab)

Belimumab was the first biologic to be used in both systemic lupus erythematosus (SLE) and lupus nephritis (LN) patients. Data from recent retrospective studies have shown that the introduction of belimumab significantly reduces glucocorticoid dependence in patients with SLE and that the effect of the dose reduction has been sustained for up to 12 months [135]. In addition, the results from a small 52-week randomized, double-blind, placebo-controlled pilot trial showed that treatment with belimumab may alleviate dermatofibrosis, but the difference was not statistically significant [136]. A new phase II clinical trial is investigating the potential of belimumab in combination with rituximab for the treatment of systemic sclerosis (SSc) (NCT03844061).

### 5.7 Anti-IL-17 mAbs (ixekizumab, brodalumab)

IL-17 is an important proinflammatory factor produced primarily by Th17 cells that plays a key role in adaptive immunity against intra- and extracellular pathogens or in the development of inflammation. Abnormally elevated levels or an imbalance of Treg/Th17 cells may lead to autoimmune diseases [137]. In a randomized trial comparing ixekizumab with ustekinumab for psoriasis in a head-to-head comparison, ixekizumab-treated patients reported greater Psoriasis Area and Severity Index (PASI) (76.5 %, p < 0.01) and static Physician's Global Assessment (sPGA) responses (52.9 %, p < 0.01) [138]. Brodalumab, another mAb targeting the IL-17A receptor, was first evaluated in a recent small-sample, single-arm phase I clinical trial in patients with diffuse cutaneous SSc (dcSSc), which demonstrated alleviation of dermatofibrosis compared with baseline [139]. An ongoing phase III clinical trial (NCT03957681) will help to further the understanding of the impact of targeting IL-17 for SSc.

### 5.8 Anti-IL-6R mAb (tocilizumab)

A phase III open-label trial revealed no significant difference in the mean change in the modified Rodnan skin score(mRSS) at the end of 48 years between patients in the tocilizumab group and those in the placebo group (p = 0.10), but researchers observed a significant improvement in the percent predicted value of forceful lung volume (FVC%) (p = 0.002) [140].The American Thoracic Society included evidence from five large global clinical trials, which showed that treatment with tocilizumab significantly slowed the mean absolute decline in FVC relative to baseline levels at the end of 24 and at the end of 48 weekends of treatment compared with that of the placebo group [141].

## 6 Conclusions

cGVHD is a complex disease characterized by substantial immune dysfunction, disability, and therapeutic toxicity, and the mortality associated with cGVHD has increased over time. The goals of cGVHD treatment are prevention of disease progression, acceleration of immune tolerance, reduction in symptomatic burden, control of objective presentation, and improvement in QoL, thereby allowing withdrawal from systemic immunosuppressive therapy without disease recurrence. Although novel drugs such as BTK inhibitors, JAK inhibitors and ROCK2 inhibitors expand treatment options, irreversible fibrotic progression and severe immune dysfunction along with serious infectious complications remain challenging. The development of mAbs that target specific lymphocyte subsets without affecting other cells is a promising strategy. Owing to their precise targeting, mAbs can increase the therapeutic potential of nonoverlapping pathways, providing therapeutic prospects for the immunocompromising, toxic and disabling effects of cGVHD to help us design "tailored" therapies for each patient. mAbs act locally while affecting the whole body, maintaining the immune response, allowing the reduction or even cessation of immunosuppressive therapy, and alleviating or even reversing organ-specific fibrosis, thereby reducing the burden of symptoms and improving QoL. The current FDA-approved drugs for treating SR-cGVHD are ibrutinib, ruxolitinib and belumosudil, but the mortality rate and its impact on QoL remain major clinical challenges. Therefore, there is an urgent need to further elucidate the pathogenesis of GVHD and identify new therapeutic targets. For refractory or relapsed patients, there is a need to develop drug combination models of mAbs and mAbs or mAbs and immunosuppressant therapy to repair and modulate the immune system.

There are several limitations to the use of mAb drugs in cGVHD. Owing to their inherent affinity and limited biological activity, these drugs may not be able to fulfill the therapeutic needs of cGVHD in some cases. In addition, the individualized nature of cGVHD makes it possible for efficacy to vary from patient to patient. mAb drugs often need to be used in combination with other immunosuppressants because of their single site, and the combination may increase the risk of drug‒drug interactions. In addition, mAbs themselves have side effects such as infusion reactions, immunogenicity, and long-term risks such as infections or secondary malignancies, limiting their further use. The fact that these drugs are mostly administered by intravenous infusion or subcutaneous injection, which results in poorer patient compliance than oral formulations, as well as a complex preparation process, stringent preservation conditions, and high price, make their use in the clinical management of patients with cGVHD even more difficult.

In addition, autoimmune diseases are caused by misdirected attacks by the immune system on one's own tissues, whereas cGVHD is caused by the attack of recipient tissues by transplanted donor-derived immune cells. The goal of treatment for autoimmune diseases is usually to suppress abnormally activated autoimmune responses, alleviate symptoms, and prevent disease progression, whereas the goal of cGVHD control is to modulate donor immune cell attacks on recipient tissues while maintaining adequate immune function to prevent infection and graft rejection. The effects of the mAb drugs listed above in autoimmune diseases need to be comprehensively understood when they are introduced into cGVHD studies. Under the premise of continuing in-depth mechanistic studies, clinical trials should be designed scientifically, phase I studies on safety and efficacy should be improved, and the standard of toleration should be strictly controlled.

Therefore, it is necessary to continue to search for and validate molecular targets closely related to the pathogenesis of cGVHD and improve antibody engineering technology to optimize the affinity, specificity, stability and other properties of mAbs. In addition, it is necessary to formulate personalized therapeutic regimens according to the specific conditions of patients and to conduct in-depth research on the combination of mAbs with traditional drugs or emerging therapies (other mAbs, cytokine therapy, cellular therapy) to optimize the therapeutic effect and reduce the cost of interventions for multiple pathological aspects of cGVHD. For refractory or recurrent patients, drug combination models and in-depth pharmacokinetic studies are needed to repair and modulate the immune system. Combined with biomarker monitoring and standardized follow-up, individualized treatments based on pathophysiology, staging, involved organs, and subtypes of cGVHD are needed.

We look forward to further development of mAbs for the prevention and treatment of cGVHD and believe that more effective mAb drugs will enter multicenter, large-sample randomized controlled clinical trials in the future, and mAb drugs based on preclinical studies of other potential targets will be introduced, resulting in better therapeutic outcomes and QoL in cGVHD patients.

## Data sharing statement

The datasets supporting the conclusions of this article are included within the article.

## Funding

This work was supported by the 10.13039/501100001809National Natural Science Foundation of China (82,020,108,004, 82,100,235, and 82,100,226), the National Key R&D Program of China (2022YFA1103300, 2022YFA1103304), the 10.13039/501100005230Natural Science Foundation of Chongqing (CSTB2022NSCQ-MSX1060), and the Special Project for Talent Construction in Xinqiao Hospital (2022YQB004).

## Data availability

None.

## Ethics requirements

None.

## CRediT authorship contribution statement

**Shiqin Huang:** Writing – original draft, Investigation, Formal analysis, Data curation. **Xianjing Cheng:** Investigation, Data curation. **Guancui Yang:** Investigation, Data curation. **Ruihao Huang:** Investigation, Data curation. **Yimei Feng:** Investigation, Data curation. **Lingyu Zeng:** Writing – review & editing, Supervision. **Tao Wu:** Writing – review & editing, Supervision. **Qingxiao Song:** Writing – review & editing, Supervision. **Xiaoqi Wang:** Writing – review & editing, Supervision. **Xi Zhang:** Validation, Supervision, Funding acquisition, Conceptualization.

## Declaration of competing interest

The authors declare that they have no conflict of interest.
